# Identification and characterization of circular RNAs in Qinchuan cattle testis

**DOI:** 10.1098/rsos.180413

**Published:** 2018-07-25

**Authors:** Yuan Gao, Mingli Wu, Yingzhi Fan, Shipeng Li, Zhenyu Lai, Yongzhen Huang, Xianyong Lan, Chuzhao Lei, Hong Chen, Ruihua Dang

**Affiliations:** Shaanxi Key Laboratory of Molecular Biology for Agriculture, College of Animal Science and Technology, Northwest A&F University, Yangling 712100, Shaanxi, People's Republic of China

**Keywords:** circRNAs, testis, Qinchuan cattle

## Abstract

Circular RNA (circRNA) is a new class of non-coding RNA that has recently attracted researchers' interest. Studies have demonstrated that circRNA can function as microRNA sponges or competing endogenous RNAs. Although circRNA has been explored in some species and tissues, the genetic basis of testis development and spermatogenesis in cattle remains unknown. We performed ribo-depleted total RNA-Seq to detect circRNA expression profiles of neonatal (one week old) and adult (4 years old) Qinchuan cattle testes. We obtained 91 112 596 and 80 485 868 clean reads and detected 21 753 circRNAs. A total of 4248 circRNAs were significantly differentially expressed between neonatal and adult cattle testes. Among these circRNAs, 2225 were upregulated, and 2023 were downregulated in adult cattle testis. Genomic feature, length distribution and other characteristics of the circRNAs in cattle testis were studied. Moreover, Gene Ontology and KEGG pathway analyses were performed for source genes of circRNAs. These source genes were mainly involved in tight junction, adherens junction, TGFβ signalling pathway and reproduction, such as *PIWIL1, DPY19L2, SLC26A8, IFT81, SMC1B, IQCG* and *TTLL5*. CircRNA expression patterns were validated by RT-qPCR. Our discoveries provide a solid foundation for the identification and characterization of key circRNAs involved in testis development or spermatogenesis.

## 1. Introduction

The mammalian testis produces sperms and testosterone. Therefore, it plays a central role in male reproduction. Spermatogenesis is a well-orchestrated biological procedure of cellular divisions and developmental changes that occur within seminiferous tubules of testis [1,2]. Mammalian spermatogenesis can be clearly discerned into three phases, as follows: self-renewal and mitotic multiplication of spermatogonia to form spermatocytes, a meiotic division of spermatocytes to form spermatids and post-meiotic differentiation of haploid spermatids into spermatozoa [3,4]. These three distinguishable events are controlled by well-coordinated gene regulation at transcriptional, post-transcriptional and epigenetic levels [5,6]. Approximately half of the testis-specific genes express in the late stage of spermatogenesis, sperm (30%) and spermatids (21%), whereas only a few are expressed in earlier stages, such as spermatogonia (3%) and spermatocytes (4%) [7]. Rapid advancement in technologies has identified the role of non-coding RNAs, including microRNAs (miRNAs), small interfering RNAs (siRNAs), PIWI-interacting RNAs (piRNAs) and long non-coding RNAs (lncRNAs) as important post-transcriptional regulators of gene expression in spermatogenesis [8,9]. MiRNAs and siRNAs control mRNA translation and degradation either by triggering endonuclease cleavage, promoting translation repression or accelerating mRNA decapping [10,11]. PiRNAs are important in genome defence against transposable elements, which contribute to genome integrity maintenance [12,13]. LncRNAs are crucial epigenetic regulators that modulate the transcriptional status of individual mRNA genes or even the entire chromosome [14,15].

A new group of circular RNAs (circRNAs) with covalently closed-loop structures have gained attention recently as novel molecules of gene regulation as other non-coding RNAs. CircRNAs were more stable than linear RNAs under RNase R treatment because of the lack of free 5′ and 3′ ends [16]. CircRNAs were detected in viroids, viruses and tetrahymena decades ago, but they were long considered by-products of aberrant RNA splicing or splicing errors [17,18]. With technological breakthroughs in high-throughput deep sequencing, a deeper understanding of circRNAs has only recently begun to emerge. To date, the biogenesis mechanisms of circRNAs have been proposed [19,20], thereby suggesting that circRNAs can be formed by circularization of exons (exonic circRNA, ecRNA), exons and introns (exon–intron circRNA, EIciRNA), intronic sequences (circularized intron RNA, ciRNA), antisense or the intergenic region. Although the function of animal circRNAs is largely unknown, circRNAs regulated gene expression by sequestering specific miRNAs or buffering the repression of mRNA targets [21,22]. For example, the circRNA Cdr1as, also known as CiRS-7, acts as a powerful miR-7 sponge/inhibitor in the developing midbrain of zebrafish [23]. Moreover, a few circRNAs have the potential to be translated into proteins via the insertion of an internal ribosome entry site [24].

However, very few studies on the roles of circRNAs in mammalian testis development and spermatogenesis have been reported. Deep sequencing of the transcript from mouse brain and testis showed that testis had a second greatest number of tissue-specific circRNA host genes following the brain tissue [25]. Recently, a total of 15 996 circRNAs were identified, and 10 792 (67%) were first observed in human testis. Interestingly, the 1017 initially reported circRNA-forming genes are mostly related to spermatogenesis, sperm motility and fertilization [26]. In cattle, a relative lack of abundance of circRNAs has been observed, except for the recent evidence found in bovine mammary gland [27]. A total of 4804 and 4048 circRNAs were identified in the cow mammary gland on days 90 and 250 postpartum, respectively.

To date, our knowledge of the expression status of circRNAs in the cattle testis is very limited. To investigate the relationship between circRNAs and cattle testis development and spermatogenesis, we performed ribo-depleted total RNA-Seq to detect circRNA expression profiles of neonatal (one week old) and adult (4 years old) Chinese Qinchuan cattle testes. The genomic feature, length distribution and other characteristics of the circRNAs in testis were also studied. By comparing the circRNA expression profiles from two developmental stages, we identified differentially expressed circRNAs. We performed Gene Ontology (GO) annotation and KEGG enrichment analysis for the host genes of differentially expressed circRNAs. CircRNA expression patterns were extensively validated by RT-qPCR. Our data indicated that circRNAs likely play an important role in testis development and spermatogenesis.

## 2. Material and methods

### 2.1. Sample collection

Whole testes were removed from one Qinchuan cattle at one week (neonatal) and 4 years of age (adult). The testis samples were immediately preserved in liquid nitrogen until use and sent to Gene Denovo Co. (Guangzhou, China) for sequencing in dry ice.

### 2.2. RNA preparation and RNase R treatment

Total RNAs were extracted from testis tissue using Trizol (Takara, QD), according to the manufacturer's protocol. Then, spectrophotometrical quantification was performed by NanoDrop (Wilmington, DE). For deep sequencing, the total RNAs were treated with RNase R (RNA07250, Epicenter) to degrade the linear RNAs and were purified using RNeasy MinElute Cleanup Kit (Qiagen).

### 2.3. cDNA library construction and sequencing

A strand-specific library was constructed using VAHTS Total RNA-Seq (H/M/R) Library Prep Kit for Illumina following the manufacturer's instructions. Ribosome RNA was removed for retain circRNAs. The enriched circRNAs were fragmented by using fragmentation buffer and reverse transcribed into cDNA with random primers. Second strand cDNA was synthesized by DNA polymerase I, RNase H, dNTP (dUTP instead of dTTP) and buffer. Next, the cDNA fragments were purified with VAHTSTM DNA Clean Beads, end repaired, poly(A) added, and ligated to Illumina sequencing adapters. Then, UNG (uracil-*N*-glycosylase) was used to digest the second-strand cDNA. The digested products were purified with VAHTSTM DNA Clean Beads, PCR amplified and sequenced using Illumina HiSeqTM 2500.

### 2.4. Sequence mapping and circRNA prediction

All clean reads were obtained by excluding low-quality sequences (more than 50% of low quality (*Q*-value ≤ 20) bases) or reads with more than 10% of unknown nucleotides (N) and by sequencing adapters. The clean reads were aligned to ribosome RNA (rRNA) database using short reads alignment tool Bowtie2 [28]. The rRNA-removed reads of each sample were mapped to reference genome (*Bos_taurus*_UMD_3.1.1) by TopHat2 (v. 2.0.3.12) [29]. Twenty mers from both ends of the unmapped reads were extracted and aligned according to the reference genome to find unique anchor positions within the splice site. Anchor reads that aligned in the reversed orientation (head-to-tail) indicated circRNA splicing and were then subjected to find_circ [21] to identify circRNAs. CircRNAs were blasted against the circBase [30] for annotation. Those that could not be annotated were defined as novel circRNAs. The identified circRNAs were subjected to statistical analysis of type, chromosome distribution and length distribution.

### 2.5. Expression profiling and analysis of differentially expressed circRNAs

To quantify circRNAs, back-spliced junction reads were scaled to RPM (reads per million mapped reads). The RPM method can eliminate the influence of different sequencing data amount on the calculation of circRNA expression. To identify differentially expressed circRNAs across samples or groups, the edgeR package (http://www.rproject.org/) was used. We identified circRNAs with a fold change greater than or equal to 2 and a *p*-value < 0.05 in a comparison between samples or groups as differentially expressed circRNAs.

### 2.6. Gene Ontology and KEGG pathway enrichment analysis

We conducted a functional enrichment analysis of source genes to study the main functions of these source genes of circRNAs. GO enrichment analysis provides all GO terms that were significantly enriched in source genes compared with the genome background and filters the source genes that correspond to biological functions. Firstly, all source genes were mapped to GO terms in the Gene Ontology database (http://www.geneontology.org/). Gene numbers were calculated for every term. Significantly enriched GO terms in source genes were defined by the hypergeometric test in comparison with the genome background. The calculated *p*-values underwent false discovery rate (FDR) correction, with FDR ≤ 0.05 as the threshold. GO terms meeting this condition were defined as significantly enriched GO terms in source genes.

Pathway-based analysis helps further elucidate the biological function of genes. KEGG is the major public pathway-related database [31]. Pathway enrichment analysis identified significantly enriched metabolic pathways or signal transduction pathways in source genes compared with the whole genome background. The calculated *p*-value underwent FDR correction, with FDR ≤ 0.05 as a threshold. Pathways meeting this condition were defined as significantly enriched pathways in source genes.

### 2.7. Experimental validation of circRNAs

To confirm individual circRNA, RNA was reverse transcribed using random hexamers with FastQuant RT kit (with gDNase) (TIANGEN) according to the manufacturer's protocol. PCR products were gel extracted and Sanger sequenced. qPCR was performed on a BioRad CFX96 real-time PCR machine using SYBR Premix Ex Taq II (Takara, Dalian, China). Each 15 µl real-time RT-PCR reaction included 7.5 µl SYBR Green Real-time PCR MasterMix, 1 µl cDNAs and 0.4 µl primers. PCR conditions consisted of one cycle at 94°C for 5 min, followed by 39 cycles at 94°C for 40 s, 60°C for 30 s and 72°C for 30 s, with fluorescence acquisition at 74°C in single mode. Relative expression level was determined using the 2^−ΔΔCt^ method with β-actin as the control. Total RNA (10 ng) was incubated with 20 U RNase R (Epicentre) in 1× RNase R buffer at 37°C for 30 min and then extracted using acid phenol–chloroform.

### 2.8. Statistical analysis

Data are shown as mean ± s.e.m. with each measurement repeated thrice. Results were assessed for statistical significance using Student's *t*-test for normally distributed data or Mann–Whitney *U*-test for non-normally distributed data from Prism v. 6 software (GraphPad Software, La Joya, CA, USA). *P*-values of less than 0.05 were considered significant: *p < 0.05; **p < 0.01.

### 2.9. MiRNA target site analysis

To further investigate the functional roles of miRNA, putative targets of miRNAs were predicted by the combination of three softwares: namely, Mireap, Miranda (v. 3.3a) and TargetScan (v. 7.0).

## 3. Result

### 3.1. Overview of circRNA profiles in neonatal and adult cattle testes

**Figure 1.**
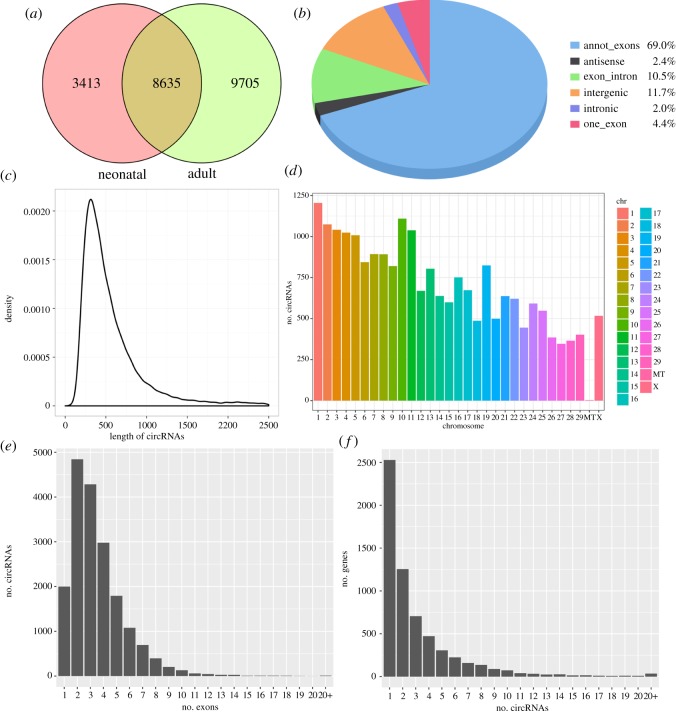
General characteristics of circRNAs in cattle testis. (*a*) Venn diagram showing overlap of annotated circRNAs among neonatal and adult cattle testis. (*b*) Distribution of genomic regions from where the detected circRNAs were derived. (*c*) Length distribution of cricRNAs. (*d*) Chromosomal distribution of circRNAs. (*e*) Distribution of exons found within exonic circRNAs. (*f*) Distribution of the number of circRNAs per gene.

To examine the circRNA expression profiles of the cattle testis during post-natal development, we profiled circRNAs in the testes from neonatal (one week old, N) and adult (4 years old, A) cattle. Ribo-depleted total RNA-Seq libraries are prepared and sequenced with paired-end 125 nucleotide (nt) reads. We decided to sequence at very high depth. We obtained 91 112 596 and 80 485 868 clean reads from two testis tissues, which could be mapped to the bovine genome at the neonatal and adult stage, respectively. A total of 21 753 candidate circRNAs were identified by at least one read spanning a head-to-tail splice junction; 12 048 and 18 340 circRNAs were identified from neonatal and adult testes, respectively. Of all circRNAs, 8635 (39.7%) were expressed in all testes (figure 1*a*). Based on their location on the genome, these 21 753 circRNAs were classified into six types, which are one_exons (4.4%), annot_exons (69.0%), exon_intron (10.5%), intronic (2.0%), intergenic (11.7%) and antisense (2.4%) (figure 1*b*). The lengths of most circRNAs (about 75.4%) were no more than 1000 nt, and the median length was 400 nt (figure 1*c*). According to their host gene location, the 21 753 circRNAs were widely scattered on all chromosomes, including the mitochondrial genome (figure 1*d*). Chromosomes 1–5 and 10–11 produced the most circRNAs (more than 1000). Most other chromosomes generated hundreds of circRNAs, except for the mitochondrial genome (only three circRNAs). Most exonic circRNAs (98.8%) were composed of no more than 10 exons, among which 16 977 (91.2%) contained one to six exons and 1632 had seven or more exons (figure 1*e*). One host gene may produce several different circRNAs. There were 2528 (41.0%) circRNA-producing genes that generated a single circRNA, whereas 93.7% produced no more than eight (figure 1*f*).

### 3.2. Analysis of differentially expressed circRNAs between neonatal and adult cattle testes

**Figure 2.**
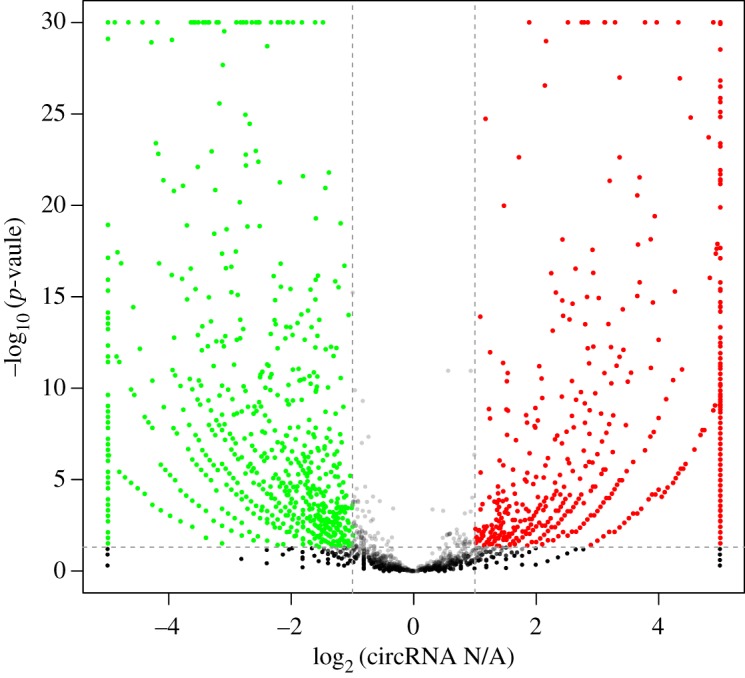
Differential circRNA expression between neonatal and adult cattle testis. Volcano plots showing –log_10_ (*p-*value) versus log_2_ fold difference in circRNA abundance in RPM between neonatal and adult cattle testis. Red circles denote significantly upregulated circRNAs, whereas green circles denote significantly downregulated circRNAs (*p *< 0.05 and fold change ≥2).

Expression profiling was performed to identify circRNAs that were differentially expressed during two developmental stages. CircRNA abundance was quantified as RPM, and differential significance between samples was calculated. According to the circRNA expression profiles, 4248 circRNAs were differentially expressed (absolute fold change greater than or equal to 2 and *p* < 0.05) between neonatal (N) and adult (A) cattle testes (electronic supplementary material, table S1). Based on the expression levels of circRNA in paired samples (adult to neonatal ratio, A/N), 2023 circRNAs were downregulated and 2225 circRNAs were upregulated in the adult testis (figure 2).

### 3.3. Gene Ontology and KEGG pathway enrichment analysis

**Figure 3.**
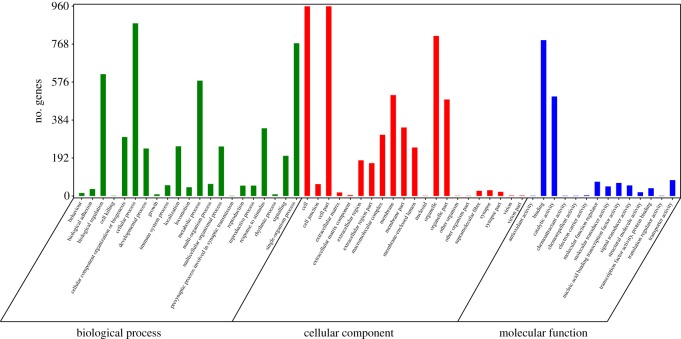
GO analysis of differentially expressed circRNA host genes between neonatal and adult cattle testis. The differentially expressed circRNA host genes are classified into the following categories: cellular component, molecular function and biological process. The left axis shows the numbers of genes.

As previously mentioned, circRNAs are most commonly generated by back-splicing events from exons of protein-coding genes. To some extent, the function of circRNAs is reflected through their host genes. Therefore, gene ontology term analysis on the host genes may provide insight into the function of circRNAs. Several GO terms significantly enriched on host genes are shown in figure 3. We submitted a list of 2328 host genes associated with differentially expressed circRNAs to the Database for Annotation, Visualization and Integrated Discovery (DAVID) for GO term analysis. The GO annotation indicated that the host genes were involved in many biological processes, such as cellular process, single-organism process, biological regulation, metabolic process, developmental process and reproduction. The main functional groups of host genes are a cell, cell part, organelle and membrane in the cellular component, and catalytic activity and binding in the molecular function.

Based on the KEGG pathway database, a pathway analysis was performed to predict the significantly enriched metabolic pathways and signal transduction pathways in the host gene of differentially expressed circRNAs. After pathway enrichment analysis, the significantly enriched pathways are shown in electronic supplementary material, table S2 (*p* < 0.05). In total, 679 host genes had KEGG pathway annotations. The significant signalling pathways included 47 pathways, including tight junction, adherens junction, TGFβ signalling pathway, progesterone-mediated oocyte maturation and oocyte meiosis.

### 3.4. Experimental validation of predicted circRNAs in neonatal and adult cattle testes

**Figure 4.**
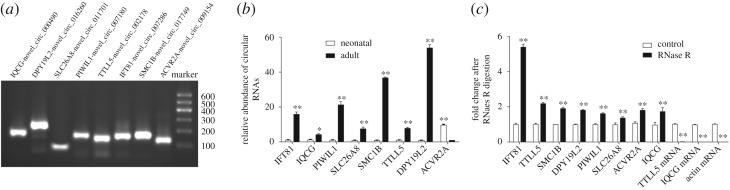
Validation and RNase R resistance of circRNAs. (*a*) Electrophoretogram for circRNAs in RT-PCR. (*b*) RT-qPCR validation of circRNA expression changes between neonatal and adult cattle testis. RT-qPCR data were calculated by the 2^−ΔΔCt^ method with β-actin as an internal control. The data are presented as the mean ± s.e.m.; **p < 0.01, *p < 0.05. (*c*) CircRNAs were resistant to RNase R digestion compared with linear isoforms. Input RNA was a mixed sample of neonatal and adult cattle testis. Total RNA was treated with RNase R or a mock treatment. The fractions of linear and circular isoforms were normalized to the value measured in the mock treatment, respectively.

To validate whether the differentially expressed circRNAs discovered through the sequencing were bonafide, exonic circRNAs with potentially significant and differential expression were selected for validation by qRT-PCR. From these predicted circRNAs, eight circRNAs of different abundance and lengths were randomly selected (electronic supplementary material, table S3), and divergent primers were designed for circRNA detection (electronic supplementary material, table S4). The eight host genes are piwi-like RNA-mediated gene silencing 1 (*PIWIL1*), dpy-19-like 2 (*DPY19L2*), solute carrier family 26 member 8 (*SLC26A8*), intraflagellar transport 81 (*IFT81*), structural maintenance of chromosomes protein 1B isoform X2 (*SMC1B*), IQ motif containing G (IQCG), tubulin polyglutamylase TTLL5 isoform X1 (*TTLL5*) and activin A receptor type IIA (*ACVR2A*). RT-PCR showed that all the eight circRNAs could be detected (figure 4*a*). Sanger sequencing confirmed the predicted head-to-tail splice junctions (electronic supplementary material, figure S1). Real-time quantitative RT-PCR revealed seven upregulated and one downregulated expressed circRNAs as shown in figure 4*b*, which clearly showed that expression patterns of these eight selected circRNAs were concordant with RNA-Seq results. These results demonstrated that the ribo-depleted total RNA-Seq results were reliable.

RNase R, a 3′ to 5′ exoribonuclease, can digest linear RNAs with their circular isoforms remaining intact. Compared with linear RNAs, circRNAs are generally endowed with a strong resistance to exonuclease RNase R with few exceptions [16]. In our study, these testis-derived circRNAs showed resistance to RNase R digestion compared to the linear mRNA controls (figure 4*c*). We treated total RNA with RNase R. Then, we performed cDNA synthesis and qPCR. All 8/8 tested circRNAs were more resistant to RNase R than the linear mRNA controls, of which 2/2 were highly degraded by RNase R. After RNase R digestion, the expression of eight circRNAs did not obviously decrease, but most of them notably increased. We speculated that the enhancement of amplification efficiency might result from the relative amount of random primers and increased dNTPs after linear RNA depletion in the reaction system. RNase R digestion not only relatively increased the concentration but also ensured the purity of circRNAs, especially when some mRNA fragments are part of circRNA loops. Moreover, resistance to RNase R supports the prediction of circRNAs. Thus, RNase R digestion is essential for the detection of circRNAs.

### 3.5. The target miRNAs of differentially expressed circRNAs in neonatal and adult cattle testes

Over the last few years, some reports have indicated that miRNAs were involved in all aspects of spermatogenesis and testis development [10,11]. Thus, they can be used as biomarkers for male infertile diseases. Recent studies of two individual circRNAs suggested that circRNAs act as miRNA sponges and play an important role in regulating gene expression through a circRNA–miRNA–gene pathway [21,22]. MiRNA prediction was performed via the combination of three softwares: Mireap, Miranda (v. 3.3a) and TargetScan (v. 7.0). A total of 4244 differently expressed circRNA target miRNAs were found (electronic supplementary material, table S5). A total of 758 miRNAs were predicted, and some of them have been reportedly associated with spermatogeneses, such as bta-miR-532, bta-miR-204 and bta-miR-34 family [32,33].

## 4. Discussion

In this study, we performed the first demonstration of testis development and spermatogenesis for circRNAs in cattle testis. Using exceptionally deep total RNA-Seq data, we identified 21 753 circRNAs in neonatal and adult cattle testis. The number of identified circRNAs is much more than that obtained in a previous study involving the mammary gland [27]. The majority length of circRNAs is more than 1000 nt, and the median length was 400 nt; these values are similar to previous observations on human testis samples [26]. These observations in cattle testis combined with previous findings in humans might indicate that length characteristic is a universal feature of testis tissues.

We found that 2225 (10.2%) of circRNAs were upregulated in adult cattle, whereas 2023 (9.3%) were downregulated (figure 2). Previous studies employed RNA-Seq to profile testis development or spermatogenesis. An analysis of transcriptomic changes in yak and cattle yak testis tissues revealed an obvious bias towards upregulation rather than downregulation [34]. In the 2960 identified differentially expressed genes, 679 were upregulated and 2281 were downregulated. In the RNA-Seq of porcine sexually mature and immature testis, 10 095 genes were significantly differentially expressed, including 242 spermatogenesis-associated genes. A total of 5199 and 4896 transcripts were upregulated and downregulated in mature testis, respectively [35]. A total of 3025 lncRNAs were differentially expressed between neonatal and adult mouse testes. A total of 1062 lncRNAs were significantly downregulated, and 1963 lncRNAs were significantly upregulated in the adult testis [36]. Some of these RNAs include protein-coding mRNAs and long non-coding RNAs, such as *PIWIL1, Wt1* and *Sycp2*. One main motivation for profiling testis transcriptomes is to uncover factors that might play a role in testis development and spermatogenesis, thereby elucidating the underlying molecular mechanism.

To further define the biological processes that involve circRNAs, we performed GO annotation and KEGG pathway analysis. These processes could point the roles for testis circRNAs or might reflect the cellular difference between neonatal and adult cattle testes. In GO annotation, 44 host genes were assigned to reproduction and reproductive process, and 25 host genes were associated with spermatogenesis, including *PIWIL1* and spermatogenesis-associated protein 6 (*SPATA6*). PIWI proteins are associated with piRNAs and play crucial roles during germ-line development [37], stem cell maintenance [38], spermatogenesis [39] and transposon regulation [40]. *PIWIL1* and its various homologs in *Drosophila* and zebrafish are required for both male and female fertility [41,42], whereas in mice, the *PIWIL1* homologue *MIWI* is essential for male fertility and appears to primarily function in the pachytene stage of spermatogenesis [39]. Spermatogenesis-associated (SPATA) genes are a large family of genes that play a very important role in testis development and spermatogenesis. The highest levels of *SPATA6* mRNA were detected in spermatids [43]. Recently, Yuan suggested that inactivation of *SPATA6* could result in male sterility and acephalic spermatozoa [44]. In our study, *PIWIL1* and *SPATA6* were upregulated in the adult cattle.

In KEGG pathway enrichment, 47 significantly enriched pathways were obtained (*p* < 0.05), including tight junction, adherens junction and TGFβ signalling pathway. The TGFβ ligand superfamily is composed of more than 40 members, most of which are produced in the mammalian testis to regulate testis development and germline differentiation [45]. In our study, a total of 16 host genes, including transforming growth factor, beta 2 (TGF*β*2), transforming growth factor, beta receptor II (TGFBR2), activin A receptor, type IIA (ACVR2A) and SMAD family member 2 (SMAD2), were involved in the TGFβ signalling pathway. Cell–cell adhesion between seminiferous epithelium is mediated by tight junctions (TJs) and anchoring junctions, such as the cell–cell actin-based adherens junctions (AJs) and cell–cell intermediate filament-based desmosomes [46]. During the entire process of germ cell development, except for the early phase of spermatogenesis from type B spermatogonia up to preleptotene and leptotene spermatocytes, it is segregated from the systemic circulation because of the blood–testis barrier (BTB) created by TJs between Sertoli cells. However, preleptotene and leptotene spermatocytes migrate progressively from the basal to the adluminal compartment of the seminiferous epithelium, thereby traversing the BTB; this event of cell movement is accompanied by an extensive restructuring of cell–cell actin-based AJs between Sertoli and germ cells [47]. Evidence suggests that TJs and AJs are the key events in the regulation of spermatogenesis [48].

In 2013, circRNAs were identified as efficient miRNA sponges [21,22]. Overexpressing miRNA target site concatamers (miRNA sponges) results in loss of miRNA function accompanied by increased endogenous target gene expression [49]. Therefore, some circRNAs may inhibit or relieve repression of miRNA on translation. In this study, 758 miRNAs matched with significantly differentially expressed circRNAs, in which the bta-miR-532, bta-miR-204 and bta-miR-34 families are shown to be involved in cattle spermatogenesis [32,33]. Additionally, the miR-34a was reportedly associated with the spermatogenesis of zebrafish; miR-34a knockout zebrafish showed no obvious defects in terms of testis morphology and sperm quantity [50]. Hence, the related circRNAs may act as miRNA sponges for regulating testis development and spermatogenesis.

This is the first study to delineate the expression profile of circRNAs in Qinchuan cattle testis. Abundant circRNAs were detected in the testis, and the genomic feature, length distribution and other characteristics of the circRNAs were revealed, thereby laying the basis for future studies on the function of circRNAs in Qinchuan cattle testis. All these discoveries provided a solid foundation for the identification and characterization of key circRNAs involved in testis development or spermatogenesis.

## Supplementary Material

Table S1 CircRNAs differentially expressed in neonatal and adult cattle testis

## Supplementary Material

Table S2 Significantly enriched pathway of differential expressed circRNAs

## Supplementary Material

Table S3 List of 8 validated testis derived circRNAs

## Supplementary Material

Table S4 Primers of 8 validated testis derived circRNAs for general and quantitive RT-PCR

## Supplementary Material

Table S5 MicroRNA target site analysis for differential expressed circRNAs

## Supplementary Material

Figure S1 Sanger sequencing map of the validated circRNAs

## Data accessibility

The full set of raw data from this study has been deposited in the National Center for Biotechnology Information's Sequence Read Archive (SRA) and is accessible through the SRA accession SRP133991 (https://www.ncbi.nlm.nih.gov/sra/SRP133991).
