# Supplementary material for: Identification and characterization of circular RNAs in Qinchuan cattle testis
Source: R Soc Open Sci. 2018 Jul 25;5(7):180413. doi: 10.1098/rsos.180413 (PMC6083711; doi:10.1098/rsos.180413)
Supplement: Table S3 List of 8 validated testis derived circRNAs [file rsos180413supp3.docx]

**Table S3 List of 8 validated testis derived circRNAs.**

| circRNA id | Chromosome | Genomic start | Genomic end | Spliced length (bp) | strand |
| --- | --- | --- | --- | --- | --- |
| novel_circ_000490 | 1 | 70796976 | 70799148 | 335 | + |
| novel_circ_002178 | 10 | 88044336 | 88045386 | 480 | + |
| novel_circ_007180 | 17 | 47742463 | 47748869 | 357 | - |
| novel_circ_007286 | 17 | 56333689 | 56345720 | 407 | + |
| novel_circ_009154 | 2 | 48411976 | 48418830 | 473 | - |
| novel_circ_011701 | 23 | 9867947 | 9882955 | 838 | - |
| novel_circ_016260 | 4 | 62265014 | 62305995 | 856 | + |
| novel_circ_017749 | 5 | 116494342 | 116519503 | 689 | - |
