# Supplementary material for: Identification and characterization of circular RNAs in Qinchuan cattle testis
Source: R Soc Open Sci. 2018 Jul 25;5(7):180413. doi: 10.1098/rsos.180413 (PMC6083711; doi:10.1098/rsos.180413)
Supplement: Table S4 Primers of 8 validated testis derived circRNAs for general and quantitive RT-PCR [file rsos180413supp4.docx]

**Table S4 Primers of 8 validated testis derived circRNAs for general and quantitive RT-PCR.**

| Gene Symbol | CircRNA ID | Forward primer | Reverse primer | Length (nt) | Tm  (℃) |
| --- | --- | --- | --- | --- | --- |
| *PIWIL1* | novel_circ_007180 | F:TGCGTAATGATTTTAATGTGATG | R:AAGGTACTCTTTGGATTCTGGTC | 166 | 60 |
| *DPY19L2* | novel_circ_016260 | F:CAGTTTTGCTTTGTTTTGTATTC | R:TCATAATCATCCATAGTCCTTGC | 244 | 60 |
| *SLC26A8* | novel_circ_011701 | F:AAAGATTTGTCGGTGTTAC | R:TGCTCATATCTGGCGTTA | 84 | 60 |
|  |  |  |  |  |  |
| *IQCG* | novel_circ_000490 | F:AGAAACAGCCAAAGAAGCAGTCA | R:AATCTCACGGAAATGCAATCACC | 191 | 60 |
| *TTLL5* | novel_circ_002178 | F:CGCTGTCGATCAGGCAGTTAC | R:TGCTGTTTGGGCTGCTTTATT | 152 | 60 |
| *IFT81* | novel_circ_007286 | F:CCGTGAATATGACGGCACT | R:ATAGCAGGCTCTGAAACCA | 170 | 60 |
| *SMC1B* | novel_circ_017749 | F:GAACAAGCATATTAAACAGCAAC | R:CTTCCAGAGTCTCACAGACAAGG | 181 | 60 |
| *ACVR2A* | novel_circ_009154 | F:CCTGTGCTCGTTCCAACTCAA | R:AATGCCGCCGTTTATCTTTGT | 144 | 60 |
| *TTLL5*-mRNA |  | F:AACTGGAGGAGGTGTTGACTT | R:CTGTTTGGGCTGCTTTATTTT | 167 | 60 |
| *IQCG*-mRNA |  | F:TATTGCTCACCTCAAGGACCA | R:TTCTTCAGTTTTCAGCCGTAG | 172 | 60 |
| *β-actin* |  | F:GCGTTACACCCTTTTTCTTGACA | R:TCACCTTCACCGTTCCAGTTT | 152 | 60 |
