# Supplementary material for: Identification and characterization of circular RNAs in Qinchuan cattle testis
Source: R Soc Open Sci. 2018 Jul 25;5(7):180413. doi: 10.1098/rsos.180413 (PMC6083711; doi:10.1098/rsos.180413)
Supplement: Figure S1 Sanger sequencing map of the validated circRNAs [file rsos180413supp6.docx]

**
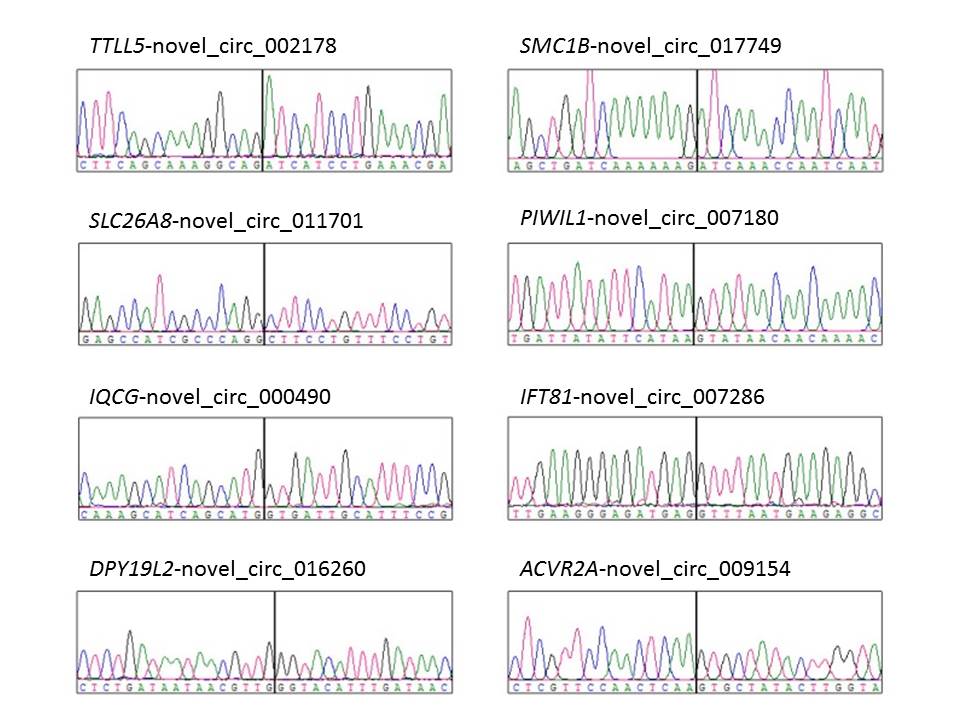
**

**Figure S1 Sanger sequencing map of the validated circRNAs.**

Black vertical line: splicing junction.
